# Ethical Treatment of Invasive and Native Fauna in Australia: Perspectives through the One Welfare Lens

**DOI:** 10.3390/ani12111405

**Published:** 2022-05-30

**Authors:** Brooke P. A. Kennedy, Nick Boyle, Peter J. S. Fleming, Andrea M. Harvey, Bidda Jones, Daniel Ramp, Roselyn Dixon, Paul D. McGreevy

**Affiliations:** 1 School of Environment and Rural Science, University of New England, Armidale, NSW 2351, Australia; pmcgree2@une.edu.au; 2 Taronga Conservation Society Australia, Bradleys Head Road, Mosman, NSW 2088, Australia; nboyle@zoo.nsw.gov.au; 3 Vertebrate Pest Research Unit, NSW Department of Primary Industries, Orange Agricultural Institute, 1447 Forest Road, Orange, NSW 2800, Australia; peter.fleming@dpi.nsw.gov.au; 4 Ecosystem Management, University of New England, Armidale, NSW 2351, Australia; 5 Centre for Compassionate Conservation, TD School, University of Technology Sydney, Ultimo, NSW 2007, Australia; andreaharvey.cat@googlemail.com (A.M.H.); daniel.ramp@uts.edu.au (D.R.); 6 Sydney School of Veterinary Science, University of Sydney, Sydney, NSW 2006, Australia; bidda.jones@sydney.edu.au; 7 School of Education, University of Wollongong, Northfields Avenue, Wollongong, NSW 2522, Australia; roselyn@uow.edu.au

**Keywords:** animal welfare and ethics continuum, biodiversity, conservation, feral animals, invasive species, population control, zoos

## Abstract

**Simple Summary:**

A public forum can reveal a wide range of perspectives on the ethical treatment of animals. This article describes how a panel of experts navigated through a discussion on the many and varied challenges of attempting to manage invasive and native fauna in Australia. The panel acknowledged the variety of these fauna, their effects on others and the consequences of control measures for three parties: animals, humans and the environment. The One Welfare concept has been developed to guide humans in the ethical treatment of non-human animals, each other and the environment. The forum accepted the need to consider this triple line, and exemplifies the merits of a One Welfare approach to discussions such as this. We used a series of questions about past, present and anticipated practices in wildlife control as the core of the panel discussion. We revealed five different but intersecting perspectives: conservation action, wildlife research, invasive animal ecology, mainstream animal protection and compassionate conservation. This article shows how understanding of lines of contention on various core topics can provide a framework for further discourse that may bear fruit in the form of One Welfare solutions.

**Abstract:**

The One Welfare concept is proposed to guide humans in the ethical treatment of non-human animals, each other and the environment. One Welfare was conceptualized for veterinarians but could be a foundational concept through which to promote the ethical treatment of animals that are outside of direct human care and responsibility. However, wild-living animals raise additional ethical conundrums because of their multifarious values and roles, and relationships that humans have with them. At an open facilitated forum, the 2018 Robert Dixon Memorial Animal Welfare Symposium, a panel of five experts from different fields shared their perspectives on “loving and hating animals in the wild” and responded to unscripted questions from the audience. The Symposium’s objectives were to elucidate views on the ethical treatment of the native and invasive animals of Australia and to identify some of the resultant dilemmas facing conservationists, educators, veterinarians and society. Here, we document the presented views and case studies and synthesize common themes in a One Welfare framework. Additionally, we identified points of contention that can guide further discourse. With this guide in place, the identification and discussion of those disparate views was a first step toward practical resolutions on how to manage wild-living Australian fauna ethically. We concluded that there was great utility in the One Welfare approach for any discourse about wild animal welfare. It requires attention to each element of the triple bottom line and ensures that advocacy for one party does not vanquish the voices from other sectors. We argue that, by facilitating a focus on the ecology in the context of wild animal issues, One Welfare is more useful in this context than the veterinary context for which it was originally developed.

## 1. Introduction

Australia has a unique vertebrate fauna, of which 340 terrestrial species are in danger of extinction [1]; 60 were anthropogenically introduced, and most of these are invasive [2]. A few native birds and mammals have benefited from increased anthropogenic resources and multiplied their numbers and distribution over the past ~230 years. Some have also become invasive, for example, rainbow lorikeets (*Trichoglossus haematodus moluccanus*) in Western Australia [3] and flying foxes (*Pteropus* spp) in eastern Australian towns and cities [4]. Introduced and native animals that become invasive are particularly contentious, and even the terminology used to describe them generically is subject to debate (e.g., the use of militaristic language [5]).

The title of the 8th Robert Dixon Memorial Animal Welfare Symposium (RDMAWS), “Loving and Hating Animals in the Wild” originated from a controversial feature film that had just been released: “Kangaroo: A Love-Hate Story” [6]. Kangaroos (Macropodoidea) are just one of the groups of wild-living animals in Australia that have different levels of value among different groups of people, and their feelings toward them range from love through apathy to hate (For Australian fauna, we adhere to the taxonomy and nomenclature of Jackson and Groves [7].). At the Symposium, conservation was placed at the center of the discussion about if, when and how we should intervene in the lives of wild animals and why we should consider the consequences for their welfare.

The roles of animals in society change. Accordingly, individual perspectives and society’s expectations of how we manage and care for animals also change. Likewise, the evidence based upon which many of those expectations are laid changes with the iterative process of scientific investigation. Lay people, ethicists, veterinarians, ecologists and animal welfare and conservation scientists are all stakeholders in the discourse about the welfare of wild-living animals. It is imperative that the scientific voice is heard as an equal partner in the social debate about what happens to wild animals. It is the responsibility of scientists to be at the forefront of animal welfare practice and research, to disseminate their findings and to play a leading role in evidence-based debate. Such findings can often be uncomfortable to some interest groups, but nonetheless, the academic community has a duty to not shy away from the evidence that emerges from scientific endeavors.

The One Welfare concept [8,9] is a useful, all-encompassing and ethical framework that links animal welfare, human welfare and conservation. Animal welfare scientists increasingly contextualize their findings within a One Welfare framework that enables multidisciplinary collaboration with colleagues in agriculture, business and marketing, the social sciences, medicine, public health, environmental studies, animal studies and law. As the younger sibling of the One Health construct, One Welfare recognizes that animal welfare, biodiversity and the environment are intertwined with human wellbeing and community resilience. The One Welfare construct assumes that stakeholders are of at least of average health so that considerations can focus primarily on their welfare. This reflects the reality that animals can be in good health but nevertheless have poor welfare.

Since its inception in 2013 [9], One Welfare has gained scientific momentum with three international conferences but continues to act as the welfare-focused companion to the more established One Health strategy. The linkage of increased biodiversity with human well-being is a desired outcome of the One Welfare concept, but the welfare of wild animals themselves is not explicitly stated. Initially, One Welfare was designed for veterinarians to foster animal welfare, and human and societal well-being. The inclusion of a sustainable environment outcome means that One Welfare could be a foundational concept and would promote the ethical treatment of animals that exist outside of direct human care and responsibility.

**Figure 1:**
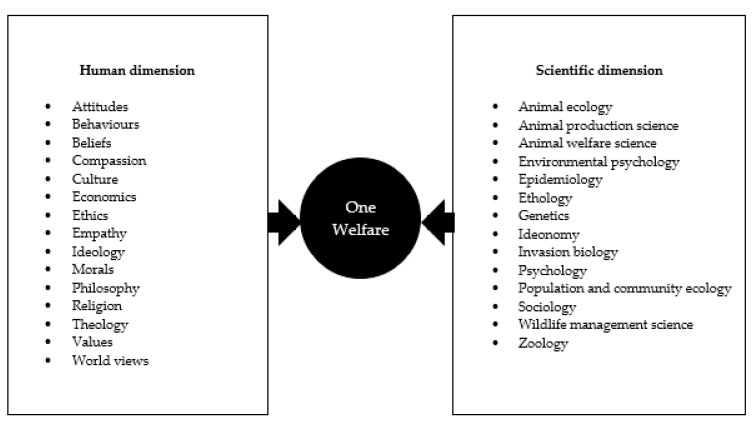
Some human and scientific dimensions that may contribute to understanding of the issues that arise in the One Welfare concept and its objectives.

The RDMAWS panelists represented five different but intersecting perspectives: conservation action, wildlife research, invasive animal ecology, mainstream animal protection and compassionate conservation. They represented some of the disciplines and worldviews that are involved in decision-making about the welfare of wild-living animals (Figure 1).

Here, we present the opinions of the five stakeholder groups who represent various voices along what we introduce as the animal welfare and ethics continuum. Along this continuum, we identify areas where approaches align and diverge, and identify points of tension among stakeholders. We identify how those voices, agreements and tensions fit within a One Welfare outcome for wild-living Australian fauna. We acknowledge that the opinions of the authors differ, and the views presented here are not unanimously held, that not all contested views were debated within the time limits of the Symposium and that other unexplored views may be held by authors. Furthermore, we acknowledge that individual authors from any given field cannot represent every view from that field.

Finally, we explore how the recently published CoMM4Unity Framework [10] aligns with our goals. The framework aims to address complex issues by involving all stakeholders, in all processes of designing and implementing solutions. We recognize the complexity of animal welfare issues in Australia involving, as it does, diverging frames (a tool that categorizes individuals’ interpretations into aggregate norms regarding the nature of the situation), differing knowledge and variable power among multiple stakeholders. The framework offers guidance in defining the issue(s) discussed herein, but also paves the way for all stakeholders to work collectively to make an impact.

## 2. Setting the Scene: The Conflict of Loving and Hating Wild Animals

We wanted to address this topic, and the first voice in the continuum was the voice of conservation action. This voice highlighted the responsibility of delivering two strategic pillars—one of them being conservation outcomes and the other being outcomes for animals in human care. These pillars include health programs and animal welfare programs. Often, these two pillars can come into conflict. Therefore, the core issue is less about loving and hating animals and more about what one ought to do, or what is the right thing to do in a given scenario. Additionally, conservation action has a responsibility for ethical decision-making processes for the treatment and care of animals, including those in the wild.

One specific example is the conservation of the Plains-wanderer (*Pedionomus torquatus*). The Plains-wanderer is a critically endangered bird species that was once common from Victoria through to Queensland, and can now only be found in scattered populations in western Victoria, eastern South Australia and around the western Riverina region in southern New South Wales, Australia. Their numbers in the wild have declined by about 90% over the last 14 years. In response, an insurance population for this species has been established at Taronga and Taronga Western Plains zoos. It was an especially difficult decision to make, as the care of Plains-wanderers in captivity was new, and the zoos had yet to acquire substantial knowledge about their care in captivity.

This decision prompted a critical question: is it right to take these birds in when the capacity and skills to care for them is not guaranteed? Ultimately, the situation was deemed urgent and the decision to act and intervene was made. However, the decision required consideration of those sorts of animal welfare and care concerns against the potential conservation outcome.

In terms of animal welfare and conservation, the topic of loving and hating animals in the wild seems to suggest an emotional response. Indeed, conservation itself is a highly emotional vocation, in that our moral concern and empathy for species may be driven by our emotional response to decline anthropogenic impact. The second voice on the continuum is that of mainstream animal protection, and usually takes a scientific standpoint and steps back from the issue to consider the evidence. Although emotion and reason are often viewed as being in conflict with one another, philosophically they are commonly considered as linked and complementary [11]. Traditionally, animal protection would advocate from the animal’s perspective. However, there can be different points of view when considered in terms of human–animal conflict.

One example of this could be drawn from a family lucky enough to live in a conservation area, protected under the NSW Government’s Biodiversity Conservation Trust. The home’s clearing is fenced, but there is currently a resident wombat (*Vombatus ursinus*) that has broken through this fence every single night for two months. Other wombats came into the clearing before, but it usually took only a couple of weeks of fixing the fence until they stopped. However, this wombat was persistent in his desire to be on the inside of the fence. Subsequently, the family spent about 50 h outside on their hands and knees in temperatures as low as −2 °C twisting wire and digging, just trying to deal with the damage caused by the wombat. Over that time, the family may have harbored severe ill-will towards the wombat, despite being on the side of mainstream animal protection.

Such an experience illustrates that our emotional response to wild animals can cause considerable personal angst and can shape the way decisions are made. Indeed, a reason why we have so much conflict with wildlife in Australia is because of the extensive costs to people’s time and resources in dealing with their direct effects. This is why some people tend to demonize certain species and assign them labels such as “vermin” and “pests”. Additionally, it is an argument some people use when they attempt to justify inhumane treatment of wild animals.

Adding to this point of emotional implications in loving and hating animals, wildlife research was the third voice on the continuum. This voice cited the case of wild horses (*Equus caballus*) as a good example in which one can note extreme views. Very often, these human–animal conflicts result from the situation that an animal finds itself in. Thus, the point of the conflict might not necessarily be an emotional view of that particular species, but an emotional view for the species’ situation.

With wild horses, there is certainly a broad spectrum of views that people take on them. These range from a passionate belief that wild horses belong in the wild and that they should be protected to the belief that they should not be in the wild [12]. Additionally, there are many views in between, including beliefs among individuals who love horses but who also agree that there are some places that wild horses should not be.

It is important to note that the process of people categorizing themselves as lovers or haters of a species does not assist in resolving the conflict. Certainly, in the wild horse debate in Australia, stakeholders are often labelled as pro-brumby or anti-brumby. This does not resolve the conflict because these matters are often extremely complex. Such categorization does not address the reasons why people have such different emotional responses to particular animals, nor the reasons that underpin those emotions. The voice of wildlife research would tackle this conflict by adopting approaches beyond categorizing people as loving or hating a particular species.

One such method of reaching for underlying reasons for diverse views on animals in the wild can be exemplified by the fourth voice on the continuum, that of invasive animal ecology. This voice has a role in directing research on invasive animals, their ecology and management and their interactions among themselves, having conservation effects and agricultural protection effects—in addition to the human component. The analysis of the interactions of predators, their prey, the plants that they eat and the people they interact with prompts a perspective that is neither loving, nor hating, nor apathetic towards invasive species, but which may be driven by strong connection to, and love for, native species.

Often, those involved in invasive animal ecology are placed in situations that require making value judgements and are nearly always placed in situations that require making judgements between competing values. In both work and general life, people are ethically diverse. They have different world views and ethics, and one must not be so arrogant as to think that one’s ethics should prevail over another’s. This was illustrated with an example of a culturally-based ethical difference experienced by an Australian ecologist while being driven by a colleague. The two were following a car that drove into and hit several galahs (*Eolophus roseicapilla*) foraging on the road, of which one was seriously injured but alive. Raised in rural Australia, the ecologist had been taught that when one sees an animal in distress and there is nothing that can be done to save its life, one should “put it out of its misery”—believing this the ethical and morally right thing to do. As the bird flapped helplessly around the road, it was obvious that it was going to die. From the passenger seat, the ecologist told their colleague to “run it over” to quickly end its suffering. However, the colleague purposely straddled the bird instead—an action that, from the ecologist’s ethical stance, was shocking. However, the driver’s ethics required that the galah be given a chance “to live out its natural life”, even though it was in a situation in which a human had caused it distress and pain. This situation revealed competing ethics around how an animal should be treated: neither was right or wrong; they were just different. Indeed, such competition between world views and sets of ethics is even more frequently reflected in the situation where a rabbit (*Orycyolagus cuniculus*) blinded and disabled by the effects of myxomatosis virus (introduced to control rabbit numbers in Australia [13]) is presented to veterinarians as a candidate for euthanasia on humane grounds.

The voice of invasive animal ecology posited that the notion of loving or hating invasive or native animals is irrelevant. As the voice of mainstream animal protection previously exemplified, people should always consider the welfare of the animals, even when trying to control them to preserve one particular value, regardless of one’s feelings toward the animal. However, this should be done while considering the welfare of the invasive animals that we are controlling. Whether people are excluding or killing invasive or native animals, the welfare of the targeted animals and non-target species must be considered as much as possible.

The fifth voice on the animal welfare and ethics continuum encompasses compassionate conservation. This voice emerges from a recognition that many animals are sentient, sapient and social beings, and thus should be considered moral persons [14,15]. The voice of compassionate conservation posited that to love other animals is to respect their intrinsic value and autonomy; and that to hate other animals is to sanction domination and subjugation. That is not to say that acts of hate cannot be conceived from a deep concern for those we love, as evidenced by the “violent love” enacted by many conservation programs around the world [16]. However, these acts stifle the possibilities of connecting to and coexisting with other beings as fellow persons, with compassion, humility and justice. Many cultures recognize that we are teamed up with non-human persons with whom we are closely entangled in life and in death—e.g., [17]. There is much to learn from these cultures.

Control mechanisms acting on cooperation and sociality for individuals within a species enable those individuals to get along with one another, to share resources, to reproduce and to be valued by peers [18,19]. Thus, there are strong behavioral and biological mechanisms that encourage an individual to value those around them, which promotes empathy and compassion for others. These mechanisms, and the consequent effect of valuing others, break down when the costs of devaluing others are diminished. Often this devaluation occurs when there is no direct connection to those being devalued, or when the peer benefits of those who diminish value outweigh the costs. For humans, such tendencies further diminish when the value of other species is based on their utility rather than their value as peers.

Further, the voice of compassionate conservation noted that placing value on the utility of non-human animals is a common approach founded in utilitarian ethics [20]. One might place value on non-human animals that results in a reward, whether it is financial, in the form of something to eat or otherwise. This lack of value promotes the establishment of mainstream ideas (i.e., normative constructs) that downplay empathy and compassion for non-human animals.

All voices along the continuum can agree that values and ethics among stakeholders differ. It is important to recognize that when people employ those values to make decisions, the values are often driven by normative constructs [21,22]. This drive is important for contextualizing and expediting thinking, but norms often have an overwhelming influence on decision-making, which diminishes the value of non-human animals and may even lead to hatred. For example, in the early 2000s, an individual from a background in compassionate conservation argued for the incorporation of road-killed kangaroos into population estimate statistics to the New South Wales Kangaroo Management Advisory Committee. The goal of this committee is to set sustainable annual targets for commercial killing programs. The argument was that people should consider all kinds of threats that species face when estimating sustainable quotas and risk from commercial exploitation industries. This view was dismissed, and one member of the committee expressed the view that no-one cared about kangaroos; that there were too many of them. Over the years this person had perfected a roo-bar assembly on the front of his car so that he did not have to slow down when kangaroos were on the road in front of him. He indicated that he tries to hit as many kangaroos as possible, as a form of “management control”. Clearly, this position conflicts with the default position of empathy and compassion for life. What causes this lack of concern, or this ”hate”? Where does it originate? The voice of compassionate conservation argued that these questions about disregard and hatred are the crux of the current topic and merit further discussion.

Australia and New Zealand need to address these questions. These countries must first be contextualized by their white colonization history. British colonizers massacred indigenous populations [23] and established acclimatization societies to change the human and natural landscape to something both familiar and exploitative such that they lived “on” the land rather than “in” it [24]. These early settlers were driven by the need to survive and subsequently by profit and financial survival. This is a common pattern in empire expansion throughout the world. Presently, some land managers have sustained this legacy of colonization of Australia’s landscapes for profit. Australian deforestation rates match some of the fastest rates in the world [25], and some people do not recognize the need to share space with other animals [26]. The conflicts that we have already discussed demonstrate a conundrum of sharing space with non-human animals in a profit driven society and highlight the need to discover ways to overcome this—to both belong and allow others to belong, to not compromise love for others and to address why this appears to create such dissonance.

Given this background, the questions the RDMAWS panel was asked to address were:

Do the animal welfare outcomes reflect the different values placed on different species?
How can we measure our influence on the welfare of wild animals?
Who should decide when and how wildlife should be managed?
Would myxomatosis be able to be introduced to Australia now, given the current animal welfare and ethics landscape?

The remainder of our article examines how the panelists offered five voices in their responses, notes the questions that they raised and offers a synthesis of what was learned.

## 3. Different Value Levels We Assign to Species: Do the Animal Welfare Outcomes Reflect the Different Values Placed on Different Species?

It is important to clarify what is meant by “animal welfare” before we can appraise the ethics of different decision-making processes and how values influence animal welfare. There is much confusion between the concepts of welfare and ethics. Ethics focus on what people ought to do and how they ought to behave. Concepts include making decisions regarding wildlife, if people should intervene, when they should intervene, how they should intervene and whether a particular species should be controlled or intervened with in another way.

Animal welfare is a state within the animal itself. It is essentially how the animal is feeling, which includes a combination of its physical fitness, health and how we think it is feeling mentally [27]. There are several well-defined feelings in animals that are not based on extensive evidence, and other feelings that are [27]. For example, a negative feeling would include pain, fear or hunger. Therefore, we refer to determining how an animal feels when we consider the term “animal welfare”.

The assessment of animal welfare can certainly help inform ethical decision-making, but animal welfare and ethics are not the same concept. When people consider intervening with animal populations in certain ways, or more specifically, when people consider lethal versus non-lethal management of particular populations, it is important to emphasize that a non-lethal management option might be the ethically preferred option because it is the right thing to do in the situation (e.g., [28]). Alternatively, an offending threat may need to be removed before a preferred option can be delivered (e.g., [29,30]). The intervention decision may involve taking an animal’s life, consideration of the animal’s welfare or considering how the animal is feeling (e.g., [31]).

Sometimes, our considerations are not limited to killing animals. Other activities may involve other significant changes, such as capturing and translocating them [32,33,34], or fencing an area of their habitat to prevent them from moving into a particular area or to protect them from predators [35]. We must consider how any kind of intervention affects the animals [36]. Typically, with wildlife management, people have not been successful at accurately assessing animal welfare and the effects of those kinds of interventions [27].

When considering lethal control methods and their effects on the controlled animal’s welfare, we are referring to that animal’s suffering, fear and pain before its death. As animals cannot experience feelings after death, death itself might not be a welfare issue [37]. However, this does not necessarily exonerate the perception that it is fine to kill animals if it can be performed in a painless and fearless way. This is one intersection of the animal welfare with the ethical side of the debate. Therefore, it is important to distinguish those two as separate issues and to establish how to assess animal welfare in a way that helps inform ethical decisions rather than assume what the best decision for a particular animal may be *a priori*.

The voice of conservation action added to this definition of animal welfare by describing welfare as what an animal experiences—its enjoyment, pain, fear and distress—and emphasizes that people’s values can affect and intersect with those experiences. This approach should apply to species such as the rabbit (*Oryctolagus cuniculus*) that may exist in different contexts. In Australia, there are both pet rabbits and wild rabbits that are considered pests. Further, the Columbia Basin pygmy rabbit (*Brachylagus idahoensis*) is an endangered species in an endangered breeding program, and can be compared to rabbits in laboratories. The Columbia Basin pygmy rabbits and regular rabbits are similar species that both experience pain, fear, distress and suffering, often in similar ways. However, the way that individuals or society place value on them can significantly affect their environment, their nutrition and their health. Thus, values do sometimes intersect with animal welfare.

The voice of wildlife research sought the reasons underlying why people categorized themselves as lovers or haters of animals. Similarly, we need to understand the intersection between animal welfare outcomes and the decisions people make. Additionally, the voice of compassionate conservation noted how crucial it is to comprehend why people make those decisions in the first place: What are the underlying reasons influencing why some people decide that introduced species must be killed? What are the scientific and ethical justifications, and how strongly do they warrant taking a life? What alternatives are there? These are important questions that are often inadequately answered.

Currently, in Australia, there are many wild species that experience poor welfare outcomes and poor treatment because of the values that shape people’s attitudes and actions towards them. Sometimes, people lack concern for the welfare of animals and ignore their intrinsic value. When little value is placed on the welfare of wild animals, the way they are then treated is also affected. It is crucial that, as a society, we discuss why we act in those ways and whether those actions have broad scientific and public support. We must be cautious of justifications and actions that have become entrenched through normative conservation constructs that result in the normalization of welfare harms.

It is important to recognize conservation paradigms change constantly and that norms shift as awareness of ecological complexity grows: that is the iterative nature of science and of societal norms. The voice of compassionate conservation proposed that there is growing global recognition that the science behind the recent rise of invasion biology is a discipline rife with value-laden language [5,38]. Science does not justify the millions of animals killed and harmed because of it [39,40]. For example, the science of environmental management requires exponents to examine the justifications and efficacy of the programs that they implement and inflict on wild animals. Problematically, people often devote insufficient effort to rigorous design when studying interventions [41,42]; to establishing whether the goals that were set were achieved [43,44,45]; and to assessing the welfare outcomes of animals on the receiving end of our actions (but see [46,47,48]). The voice of compassionate conservation maintained that these transgressions occur partly because people rely too heavily on norms to drive science and at the cost of their values. Thus, “so-called” invasive species are pests by default, which makes welfare harm and the taking of their lives permissible. Values are shaped by society, and we must be inclusive and transparent when determining whether harmful actions are just. If we fail to do so, we open ourselves to transgressions of fundamental human values, such as compassion and empathy.

## 4. How Can We Measure Our Influence on the Welfare of Wild Animals?

Examining the ways in which people controlled introduced animals from as early as the early 2000s reveals practitioners’ insufficient understanding of the humaneness or animal welfare consequences of the different methods used to control introduced animals [49]. Nevertheless, this question of how people currently control introduced animals was asked persistently. For example, there are numerous methods used in rabbit control, from biological control measures, such as the release of myxomatosis or rabbit hemorrhagic disease virus, to poisons such as pindone or 1080, to trapping or fumigation of warrens. However, no-one truly understood the effects these methods had on the welfare of the rabbits themselves. People were focused on whether the methods were effective in killing rabbits, rather than on which methods were the most humane (e.g., [50,51]).

As discussed previously, measuring animal welfare involves quantifying what an animal feels or experiences. It is not just about the animal’s physical state, but the behavioral response it shows and its accompanying mental or affective state. There are numerous ways to measure the effect of a control method on an animal’s welfare that facilitate comparisons between different methods and reveal which methods are the most humane [49]. This is important for those involved in invasive animal management to understand and apply in their work.

Additionally, this approach has been considered in broader terms of the management of wildlife, but it is difficult to measure the responses of wild animals without interfering in their lives. This is one of the dilemmas people face when measuring animal welfare in wildlife.

We can measure the behavioral and physiological responses of an animal, and therefore, the capacity of stressors to cause distress and pain can be assessed. However, the voice of invasive animal ecology agreed that measuring these responses can be difficult to undertake in the wild. For example, for physiological responses, the animals must wear devices, among other procedures, to enable measurement (e.g., physiological responses of sheep preyed upon by dogs [52]). Unfortunately, researchers could negatively affect the animals’ welfare during measurement of the effect of other interventions on their welfare (e.g., [53]).

There have been extensive behavioral studies of animals conducted in the field of invasive animal ecology, such as a study on feral goats in which researchers observed them for many hours and analyzed their different behaviors [54]. Animal behavioral researchers apply a structure called an ethogram to define the frequency at which animals perform certain actions, such as showing vigilance and fear, grazing, drinking and other normal behaviors [55,56]. Then, researchers observe those behaviors that are likely linked to possible stressors imposed by people, e.g., helicopter flyovers [57]. There are two important aspects that then feed into a welfare assessment: the level of the response and the duration of the response.

In a slightly different approach to assessing animal welfare, the voice of wildlife research took a step back, with the understanding that effects on welfare are always relative and that many interventions by researchers will have some effect. Thus, the question often becomes: what is an acceptable effect or an unacceptable effect? To answer this question, researchers must take as a baseline the welfare attributes of free-living wild animals that are not experiencing the intervention.

An example of this is direct observations of wild horses, and comparisons with camera-trapping to remotely acquire photographs and videos [58]. One important realization from such evidence is that when people directly observe animals, they are not always observing all animals that are present or a representative sample. As observations are easier to make in the open grassland habitats, horses that are most often physically observed tend to be from the more dominant herds of horses that are more likely to reside in those better habitats. Such horses are more likely to have better than average welfare because of access to better nutrition, and therefore, they form larger family groups and can experience more positive welfare and exhibit positive behaviors.

Often in the wild, particularly in the case of horses, it is the animals not directly observed that are struggling the most. The horses that are never observed but that are recorded on camera traps are struggling in poor habitats in which nutrition is poor. Researchers can assess physical traits such as body condition, but they can also assess other variables such as behavior, home range size, social structures and reproductive rates. However, their level of nutrition influences all of these other factors.

For example, some groups of horses that were observed by one of us (A.M.H.) over a long period deteriorated physically due to poor food availability. In good welfare states in which they were subject to good nutrition, the horses formed large herds and spent much time partaking in positive social behaviors. However, as nutrition declined, the horse groups broke-up and they dispersed, often with horses wandering around alone, a sign of social disruption. Undernutrition had greatly affected their behavior and their social structures, and so body condition scoring represents just the first step in establishing a baseline for assessing the welfare of wild animals in this example.

Additionally, it might be helpful in answering the question: is it right to intervene or not? If the animals exhibit excellent welfare, it might not be right to intervene. Conversely, for a given population, assessing horses that are surviving but not thriving and that struggle to survive for extended periods is pivotal in answering whether intervening in that population would improve welfare.

## 5. Who Should Decide When and How Wildlife Should Be Managed?

Although many groups can be considered to have roles in managing wildlife, there is much debate about who should assume each role. Should a central role be left to politicians? Should regulation be left to government departments? Should individual landholders and managers have the final say on the fate of animals on their land, and when should the public be consulted? Should policy formulation be left to the scientists and stakeholders who form the continuum presented in the current article? Although there are many factors at play, the scientific voices identified in Figure 1 should be involved, as they offer the evidence from which to derive answers.

The voice of compassionate conservation posited that society as a whole should be involved in deciding which species belong in Australia, which do not and what happens to those that do not. From this perspective, the issue of who should manage wildlife resides in creating open and transparent discourse so that evidence and values can be freely discussed, which was agreed with by all voices. One important aspect is how different stakeholders arrive at their respective environmental positions. If, for example, there is a decision to intervene and take action against wild animals, are the fundamental ethical and scientific foundations of that intervention clearly stated? What values were used to drive the decision, and what values were transgressed? How clear was the need to intervene? Was the scientific evidence readily available and specific to the case at hand, rather than being general in nature (e.g., are rabbits unwelcome in a certain context or are all rabbits a problem)? Were clear logic and critical thinking evident? These questions reveal the difficulty of finding coherent and logical justifications for why certain decisions are made, which is a problem not restricted to the topic presently under discussion.

The voice of invasive animal ecology added a political perspective to this debate. This voice noted that Australia is a democracy and that the decisions about how wild and domestic animals are managed are made through the laws that our representatives make in Parliament [59]. Further questions that must be asked in this political side of the debate relate to whether those representatives and views should be accepted as they are now or should be changed. Those are changes can be made at the ballot box, but each electorate has many people with just one vote. Therefore, no individual can expect that their particular views regarding the welfare of animals will align with those of decision-makers.

The voice of conservation action maintained that these decisions should be evidence-based. He posited that the public should listen to scientists, notably animal welfare scientists, ecologists and conservation scientists. In addition, people should understand society’s evolving thoughts around these issues. There must be a social license to operate [60,61] (in this case to cull, curate, conserve or cultivate) underpinning these decisions, and it is incumbent on our policymakers to consider all those views.

A central challenge when considering just the social aspect of the debate or society’s views is that it is often difficult to extract the relevant information. The current authors, as voices on the continuum, can attest to the difficulty of conveying robust peer-reviewed science to a broad audience. Enabling scientific institutions to conduct relevant and robust research regularly will help with disseminating information. Additionally, it will help, with much-needed longitudinal monitoring, to know when intervention is appropriate. Such monitoring is critical because, by the time people realize that there is a need for an intervention, it is sometimes too late.

The voice of wildlife research added the argument that making ethical-based decisions should not focus on pushing one’s opinion over another’s. Ethical-based decisions should consider all stakeholders in the decision-making, and the animals and other community groups. However, it is also important that the process involves informed decision-making. Many ill-fated decisions have been founded on a misunderstanding, so evidence-based decision-making is critical to the process. That said, communication barriers can obstruct attempts to convey that evidence to the public in ways that they can understand.

The voice of mainstream animal protection also agreed with this point. This voice highlighted the evidence outlining that the more controversial and popular a political issue is, the less likely it is that politicians will make good or evidence-based decisions. There is a need for evidence, particularly scientific evidence, to influence not just how we act, but to address when we should intervene and whether intervention is justified and exactly what the intervention should be (does one shoot a rabbit or feed it a virus, resulting in a slow and painful death?).

Animal welfare science is needed to measure animal welfare effects and alert people when their actions are effective. Knowing why one intervenes in the first place is often an aspect of wildlife management not fully established from the outset [44]. The impacts of interventions should be measured and used in the decision-making [45,62,63]. The voice of mainstream animal protection maintained that, presently in Australia, several wildlife management decisions are performed at a political level. As such, the current situation would benefit from an authentic evidence-based decision-making process.

The voice of compassionate conservation added that science cannot tell one “what” to do; it can only provide information regarding what one can measure. Science provides only data and some interpretation of the data. Determining actions one “ought” to take in response to that information relies on ethics and values.

Society created a situation in Australia in which acclimatization societies were established to deliberately introduce European species into the country [64,65], without any knowledge of how that decision would affect biodiversity now. In recent years, Australia has become much more mindful of the consequences of introductions. We feel that Australians should take responsibility for that situation and should not allow it to get worse. However, some stakeholders are instead continuing to add to the consequences of this flawed decision, such as by allowing further land-clearing and habitat destruction. The effects of introduced species on the landscape and native animals, and most importantly, the effects that people continue to have through anthropogenic climate change and habitat destruction, present a changing lens through which we must seriously consider conservation and animal welfare. This is an opportunity for a One Welfare approach, which unpacks the complex nature of short, mid and long-term interactions among people, animals and the environment, rather than focusing on how people might affect individual species at a single point in time.

Sometimes, people are left in a quandary by considering the welfare of one particular group of animals or one individual and encountering a trade-off in which they favor the welfare of another group of animals or people over others. Much of the decision-making involves determining which outcome should take precedence, which always results in poor outcomes for some animals. For example, one may wish to conserve one species whose conservation, particularly when it is a predator, will affect the welfare outcomes of others (i.e., its prey). This affects everything people do in wild animal management, even when they decide to do nothing, as non-intervention also has welfare outcomes. This illustrates our central argument: that the situation and its contributing factors are complex.

## 6. Would Myxomatosis Be Able to Be Introduced to Australia Now, Given the Current Animal Welfare and Ethics Landscape?

One of the most profound innovations that was rolled-out in Australia was myxomatosis, a virus introduced to control the rabbit population. Myxomatosis’s effect on rabbits is horrendous, but was not explained to the general population before its release.

The voice of mainstream animal protection shared the opinion that Australia should never again consider releasing a disease that has such a horrific effect on the species that it targets. However, the current Australian landscape is not in the same situation as it was when myxomatosis was released, nor has the release of the virus solved the problems in terms of removing rabbits from the landscape. The release of myxomatosis had a significant effect at the time, and the current rabbit population counts are much lower than they were then. Additionally, our understanding of animal welfare is different now, and the ethical considerations are much broader than they were at that time. These days, many more factors must be embraced to gain public support for releasing a control method than were considered then.

The voice of mainstream animal protection raised two issues: what the welfare outcomes for the individual animals are and whether the practices are effective in achieving the stated goals. In response, the voice of compassionate conservation asserted that for all the money spent on killing animals in Australasia and elsewhere in the world, those actions have not substantially altered the ecologies of those ecosystems by any great degree. This is often due to misconstruing actions as goals (e.g., assessing the successful completion of a fox-baiting program in terms of the number of foxes killed). This voice hoped that people will learn to be more introspective about why they make decisions in the first place. It was hoped that they would weigh the implications of why they want to take certain actions and what the outcomes might be. Ideally, they would weigh those outcomes against all the competing values, including the welfare of other species.

**Figure 2:**
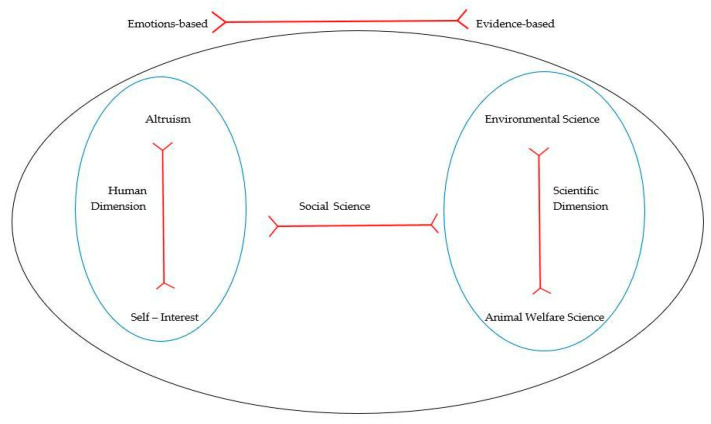
Interactions of ethical dimensions that operate when welfare is considered in wild animal management for conservation or control. The red lines represent a continuum between each alternative, e.g., between self-interest and altruism. Details of human and scientific dimensions are shown in Figure 1.

The voice of invasive animal ecology firmly maintained that myxomatosis would not be reintroduced today. This was mainly because a welfare assessment is conducted first to establish if proposed new agents are comparatively better or worse than existing methods, as part of the current selection process of any biocontrol agent. If the new agent presents low on welfare in the selection scheme, it would not be investigated further or adopted. Myxomatosis would not be accepted for introduction now because currently there are more humane biocontrols [66,67]. This neatly demonstrates how the landscape of what is acceptable in wildlife control shifts as more information comes to light and as the consequences of interventions and the ethical dimensions (Figure 2) in which voices develop are more fully understood.

Although the authors generally agree that myxomatosis should not be released today, the voice of compassionate conservation stated that there were plans to do very similar things to carp (*Cyprinus carpio*) [68], to cats (*Felis catus*) and to many other species, even knowing how traumatic death was for the affected rabbits. We are still releasing new strains of calicivirus, a hemorrhagic disease, to kill those rabbits that myxomatosis missed. We engage in these actions even when we know that the outcomes are not going to eradicate rabbits, carp or cats from Australia. All these management tools are short-term fixes that require the indefinite perpetuation of harms, with no concomitant shift in conceptualizing what belongs in Australia and what does not. Even if instantaneous and painless killing methods were developed, would mass killing be a morally acceptable action for so-called “landscape management”? Are we serving the interests of non-human animals or our own?

However, the voice of invasive animal ecology contended that the expectation of entirely “solving” an invasive species problem is unrealistic; they cannot be eradicated [69]. Additionally, the search for such a “solution” completely ignores the biological reasons for these animals being successful invaders [70,71]. Nevertheless, in most instances, instead of trying to exterminate or eliminate a particular species, people should aim to reduce the negative effects of those animals [72]. The reduction of the negative impacts upon ecosystems and other species by invasive animals may require lethal control tools to be employed [73,74].

## 7. Synthesis and Conclusions

When one considers the question of whose interests are being served in the population control measures debate, animals’ or peoples’, the two sides might not be mutually exclusive. The voice of conservation action encouraged the acknowledgement of ecosystem services and a healthy ecosystem. This approach highlights the central importance of a healthy environment and promotes human health and industry. A healthy ecosystem is one in dynamic balance where valued native species persist, rather than one that has been overrun or degraded by species that have been introduced by people.

The voice of invasive animal ecology proposed that, as a concept, invasive animal management is quite simple, and that which actions are taken depend on the situation [75]. If an introduced animal is invasive, then it will invade, take over areas and suppress other species [70,76]. The management of a detrimental invasive species would involve taking certain decisions to suppress their numbers or exclude them. If the invasive species’ effects are neutral, rather than detrimental, then nothing needs to be done. Where an invasive animal is beneficial, their numbers should be encouraged to grow. These are the basic principles that should be applied, and their welfare must be considered in each of the three situations. One should consider the negative effects and the threshold of damage and then decide what course of action should be taken [44]. The voice of conservation action agreed with this point, particularly as it applies to Australia’s unique and diverse collection of native species. Australia has the highest mammal extinction rate on Earth: 35 percent of all mammal extinctions in the last 1500 years have occurred in Australia and invasive species are the main threat to 82% of all threatened species [77]. These issues are only magnified by the island environment of Australia.

**Figure 3:**
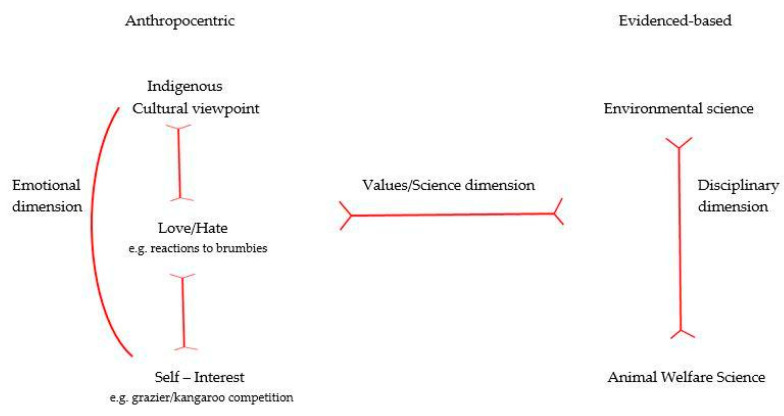
Ethical framework showing tension lines (in red) between the voices in discussions about ethical treatment of invasive and native fauna in Australia.

To address the difficulty of conveying robust peer-reviewed science to a broad audience, we have developed an ethical framework (Figure 3) that is useful for identifying the tensions between stakeholders. There is a continuum of different, but often overlapping, ethics within the human and scientific dimensions. Additionally, there are internal tensions across dimensions that are employed in decisions about the management of wild-living animals. For example, the tensions between self-interest and altruism will affect decisions about managing animals with multiple identifiable values, such as feral pigs (*Sus scrofa*) and wild horses. The different components of the framework relate to the human and scientific dimensions pertaining to a One Welfare approach, as listed in Figure 1, and to the Venn diagram showing the ethical interrelationships in Figure 2.

There is great utility in the One Welfare approach in any discourse about wild animal welfare. Presently, One Welfare has ten outcomes for human, animal and biodiversity well-being [8]. They are as follows: reduction in animal and human abuse; improved animal welfare addressing social problems; links between improved animal welfare and food safety; improved animal welfare—improved human wellbeing; more efficient multidisciplinary approaches; improved life chances—human rehabilitation and animal rehoming; improved animal and farmer welfare—improved farming productivity; improved animal welfare—addressing poverty and local community support; improved animal welfare—improved food security and sustainability; and increased biodiversity—improved human wellbeing.

These outcomes concentrate on the intertwined relationships of the welfare of farm and domestic animals and the conservation of biodiversity, along with human individual and societal well-being and reductions in things such as poverty and human and animal abuse. One Welfare requires attention to each element of the triple bottom line and ensures that advocacy for one party does not summarily dismiss or vanquish the voices from other sectors. We argue that One Welfare will facilitate a focus on the ecology of wild animals in the context of their welfare. We believe that, in this context, One Welfare will be even more useful than it already is in the veterinary context for which it was originally developed.

Since the 8th Annual Robert Dixon Memorial Animal Welfare Symposium, on which this report is based, and its subsequent national broadcast, a relevant framework that sits within One Welfare has emerged. The CoMM4Unity approach [10] was developed to purposefully enable multiple stakeholders across varying institutional levels involved in a particular complex issue to be a part of the decision-making process. Originally piloted in a remote Aboriginal community in Australia as a means of addressing an environmental dispute regarding the management of owned free-roaming dogs and cats [10,78], the framework was used to allow stakeholders with different power and knowledge levels to work together to decide on, implement and monitor steps and solutions towards a common goal [79]. The first step of the CoMM4Unity framework, “What is the issue?” identified all stakeholders (current and potential) involved in the issue.

Framing is the process of categorizing our experiences by building our current interpretations of the world around us and, in particular, in contrast to our own previous interpretations [80]. Using a systematic analysis of multiple frames for environmental disputes [80], four frames that have merged as critical and strong determinants of conflicts are: power, identity, whole-story and characterization frames. Oral histories were used to observe these frames to gain the perspectives, knowledge, values and interests of all stakeholders to define an issue, ensuring not only that all stakeholders have a voice, but also that they all agree on the issue in the first place in order to move forward with planning solutions [10]. This is the next step in determining a One Welfare approach to the complex issue of animal welfare in Australia.

The panel members in this report are stakeholders of animal welfare in Australia, all of whom fall under at least one of the voices outlined in Figure 3. Although not all voices were represented here, and the voices that were, were only represented by one author, the fact that these five voices were in the same place at the same time while endeavoring to address animal welfare is a major step. This provides the foundation of future studies where the CoMM4Unity framework may assist in the animal welfare field in Australia, not only to define the issues but to develop, implement and monitor plans together to address them.
